# Implementation research assessment instruments: a webtool to identify, compare and select assessment instruments connected with implementation science theories, models, and frameworks

**DOI:** 10.3389/frhs.2026.1855885

**Published:** 2026-07-24

**Authors:** Bryan S. Ford, Russell E. Glasgow, Kera Swanson, Rebekah Gomes, Maria Gifford, Bilquees Jamali, Ross C. Brownson, Alison Hamilton, Sara Malone, Borsika A. Rabin

**Affiliations:** 1 Adult and Child Center for Health Outcomes Research and Delivery Science (ACCORDS), University of Colorado Anschutz, Aurora, CO, United States; 2 Department of Family Medicine, University of Colorado Anschutz, Aurora, CO, United States; 3 Herbert Wertheim School of Public Health and Human Longevity Science, University of California San Diego, La Jolla, CA, United States; 4 Altman Clinical and Translational Research Institute Dissemination and Implementation Science Center, University of California San Diego, La Jolla, CA, United States; 5 Department of Physical Therapy, University of Colorado Anschutz, Aurora, CO, United States; 6 Department of Family Medicine, Data and Evaluation Team, University of Colorado Anschutz, Aurora, CO, United States; 7 Prevention Research Center, School of Public Health, Washington University in St. Louis, St. Louis, MO, United States; 8 Alvin J. Siteman Cancer Center, Washington University School of Medicine, Washington University in St. Louis, St. Louis, MO, United States; 9 Department of Psychiatry and Biobehavioral Sciences, University of California Los Angeles, Los Angeles, CA, United States; 10 School of Public Health, Washington University in St. Louis, St. Louis, MO, United States

**Keywords:** assessment, dissemination, framework, implementation, measurement, pragmatic, webtool

## Abstract

**Background:**

Implementation science theories, models, and frameworks (TMFs) are central to rigorous, theory-informed research and practice; however, linking TMF constructs to appropriate assessment instruments is challenging for many users. Existing repositories have been valuable but are fragmented, vary in accessibility, and are often limited in scope, modality, and TMF linkage. To address this gap, we expanded the Dissemination and Implementation (D&I) Models Webtool.

**Materials and methods:**

Using a user-centered, expert-informed, iterative process, we redesigned the webtool's Assess section into a public, construct-linked tool. We first identified and refined a set of instrument metadata (characteristics) through reviews of prior repositories and multistage expert engagement (subject matter experts and an external advisory board). Each candidate metadata field was rated for usefulness and feasibility, culminating in 38 finalized fields. We then purposefully selected and abstracted priority instruments to ensure diversity across modalities (quantitative, qualitative, or mixed), implementation phase, setting, audience, and equity/policy relevance. Abstraction followed a consensus approach with quality checks and regular reconciliation. Usability testing with intended users informed content, navigation, and functionality refinements.

**Results:**

Phase 1 includes 51 instruments linked to relevant TMFs and constructs. Instruments span quantitative (*n* = 33), qualitative (*n* = 14), and mixed-method (*n* = 4) formats. Common types include surveys (*n* = 31), interviews (*n* = 6), and worksheets (*n* = 3). Coverage encompasses preimplementation, implementation, and sustainment phases; varied clinical and public health settings; multiple priority user groups; and equity- and policy-relevant tools. The tool provides multipath navigation (by instrument, construct, or model), search and filtering using key metadata, and guidance for selecting and applying Implementation Science (IS) assessments.

**Conclusions:**

The Assess section of the D&I Models Webtool offers a curated, publicly available, continually updated, construct-linked tool that operationalizes TMFs through concrete measurement options across modalities and contexts. This resource supports more consistent, theory-driven assessment in IS and will continue to expand through iterative updates and community input.

## 1 Introduction

Implementation Science (IS) theories, models, and frameworks (TMFs) have been identified as one of 10 key ingredients of successful IS proposals (1). They serve as a critical backbone of IS research projects, providing guidance on refining our research questions, determining what study designs and measures we select, specifying mechanisms of change, guiding hypotheses we test, and organizing data analysis, interpretation, and presentation and dissemination of findings (2). There are many IS TMFs to choose from, with varying and often overlapping foci and constructs. Recent reviews have identified more than 150 separate TMFs (3). A meaningful use of IS TMFs requires multiple steps and includes (1) decisions around *which* TMF(s) to use, (2) potentially combining multiple TMFs, (3) adapting, and (4) operationalizing TMFs as appropriate to the study's context.

One of the most critical steps in operationalizing IS TMFs is to link them to appropriate qualitative and/or quantitative assessment approaches and instruments (1, 4). Appropriate linkage of assessment instruments to constructs in IS TMFs is essential for the systematic measurement of IS concepts across studies. However, identifying relevant assessment instruments and linking them to the IS TMF can be a daunting task, especially to those who are new to the field of IS, and failure to link a TMF to an assessment of its constructs is a frequent criticism of grant applications (2, 5–7). A structured, curated, easy-to-use inventory of both quantitative and qualitative IS assessment instruments (surveys, quantitative measures, scales, tracking forms, observational forms, etc.) would be of considerable benefit to the field and would facilitate efforts toward collecting common data elements (*NIH Common Data Elements Repository*, 2015). Historically, two measurement repositories were widely used by IS researchers—Grid Enabled Measures (GEM) and the Society for Implementation Research Collaboration (SIRC). However, these have either changed significantly or are no longer publicly accessible. Currently available assessment instrument repositories have multiple shortcomings, including not being publicly available or free, limiting their focus to a subset of IS constructs, settings, or conditions, and not allowing for direct linkage to constructs from a broad set of TMFs (1, 2, 4, 7, 8).

The development of the Assess section—now included in the D&I Models Webtool, a repository and tool to plan, select, adapt, combine, and assess D&I TMFs—was motivated not only by a specific gap in available tools but also by broader concerns about the state of IS measurement infrastructure. The field has long struggled with fragmented, inaccessible, and narrowly scoped measurement resources. The discontinuation and restructuring of widely used repositories such as GEM and SIRC has left researchers, particularly those new to the field, without a reliable, publicly available home for implementation assessment instruments. More fundamentally, even when repositories existed, they were rarely designed to connect instruments to the theoretical constructs that motivate their use. As a result, IS studies introduce TMFs in their conceptual framing but fail to operationalize those frameworks through aligned measurement, a gap that reviewers and methodologists have consistently identified as a critical weakness in the field.

The Assess section represents a step toward a different vision, one in which theory and measurement are integrated rather than parallel activities. Realizing this vision fully will require sustained community investment across several dimensions. The field needs a broader representation of instruments across underserved domains, including policy and system-level constructs, structural determinants of health, non-English instruments, and tools designed for low- and middle-income contexts. Greater standardization in how instrument characteristics are reported in primary publications would allow repositories to abstract reliable, comparable metadata rather than working around inconsistent reporting. Finally, sustainable models for shared IS infrastructure deserve explicit collective attention, as the repeated cycle of valuable resources becoming inaccessible when funding ends is a structural problem that the field has not yet solved. We invite the IS community to engage with the Assess section not only as users but also as contributors, suggesting instruments, flagging gaps, and providing feedback, so that this resource can evolve in response to the needs of the field.

Finally, a narrow range of assessments is used in IS research. Most studies use either a standardized survey that is not tailored to the study or a focus group of interview questions that may or may not be linked directly to a TMF (9).

To address this gap, the purposes of this paper are as follows: (1) describe how assessment fits into the larger Dissemination and Implementation Models in Health Research and Practice Webtool; (2) summarize the methods used to revise, restructure, expand, and update the Assess section of this webtool; (3) discuss ongoing and planned work; and (4) invite feedback and discussion to guide future directions.

**Figure 1:**
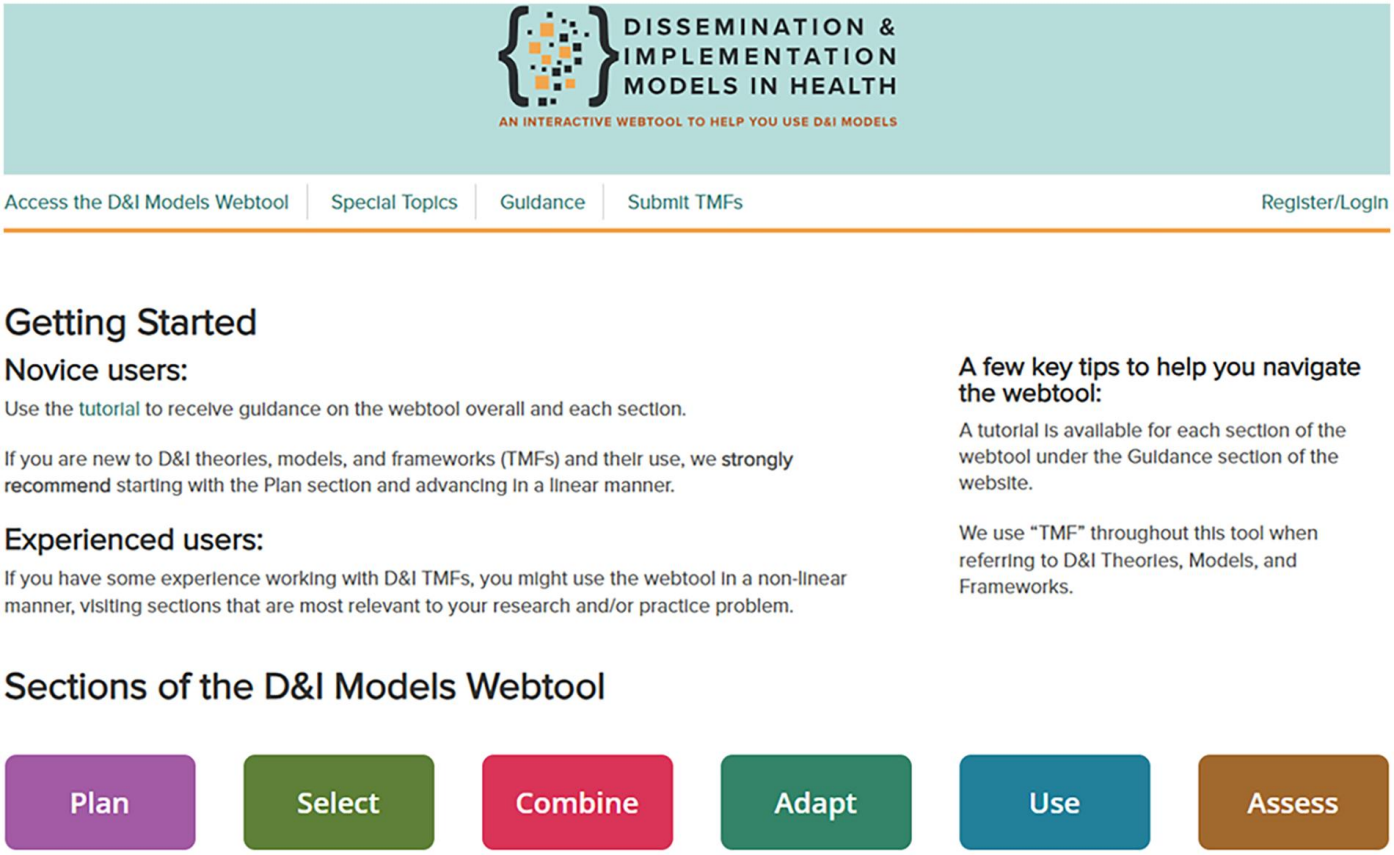
Screenshot of the D&I models Webtool.

The Dissemination and Implementation Models in Health Research and Practice Webtool (from here on referenced as the D&I Models Webtool) is an interactive webtool (dissemination-implementation.org) designed to help researchers and practitioners select the IS TMFs that best fit their research question or practice problem, combine complementary TMFs as needed, adapt the TMFs to the study or practice context, integrate the TMFs into the research or practice process, and find existing assessment instruments for the TMF constructs (10–12). These are among the most frequently asked questions and concerns from IS trainees and those new to the field (2, 7, 13). The webtool utilizes and expands upon a seminal review of IS TMFs by Tabak and colleagues in 2012. The main variables describing TMFs include the following: TMF name, primary focus on dissemination and/or implementation activities, construct flexibility, inclusion of socioecological levels, researcher and/or practitioner focus, number of times cited, primary reference(s), and examples of applications of the model. The interactive D&I Models webtool includes six main sections: Plan, Select, Combine, Adapt, Use, and Assess (formerly Measure) (see Figure 1).

### 1.1 Description

The webtool currently includes 114 IS TMFs. The initial set of models were selected from seminal articles by Tabak and colleagues (2) and Mitchell and colleagues (14) and were further expanded with additional models from more recent reviews (3, 15) and recommendations from colleagues. A network of IS scientists, including some model developers, conducted the coding of TMF characteristics (2). We also abstracted constructs and subconstructs from each TMF, allowing for keyword search. When additional TMFs were added to the webtool, we used the same standard approach for coding and abstraction (11). Crowdsourced rating and commenting functions have also been developed and made available, allowing users to provide feedback on the TMFs. Over the past 12 months (between April 2025 and April 2026), a total of 61,049 users and 60,859 sessions were documented in our usage statistics, with users from around the United States, Asia, Europe, and other parts of the world. This is an increase of approximately 25,000 users from the previous year. The webtool is considered one of the most frequently used resources in the field of IS (11, 12).

Although a section of the webtool was dedicated to linking TMF constructs (e.g., dissemination, adaptation, context, and reach) to assessment instruments from the beginning, it was a limited exercise, mostly linked to quantitative instruments and was exclusively based on linkage to external measure databases where support priorities shifted and became unavailable. Furthermore, detailed guidance on how to select and use assessment instruments—like the Select, Combine, Adapt, and Use sections—was not developed. In the following, we provide an overview of the primary objective of refining and expanding the Assess section of the D&I Models Webtool to serve as a tool linking key IS TMF constructs with implementation assessment instruments. We describe the process of selecting assessment instruments and their characteristics for this phase 1 version of the Assess section. Our secondary objective is to provide a variety of types and modalities of assessment methods in our tool to encourage both mixed-methods research and innovation in assessment in IS. We also summarize the key characteristics of the assessment instruments included and propose the next steps for advancing the field.

## 2 Materials and methods

### 2.1 Overview and funding

**Figure 2:**
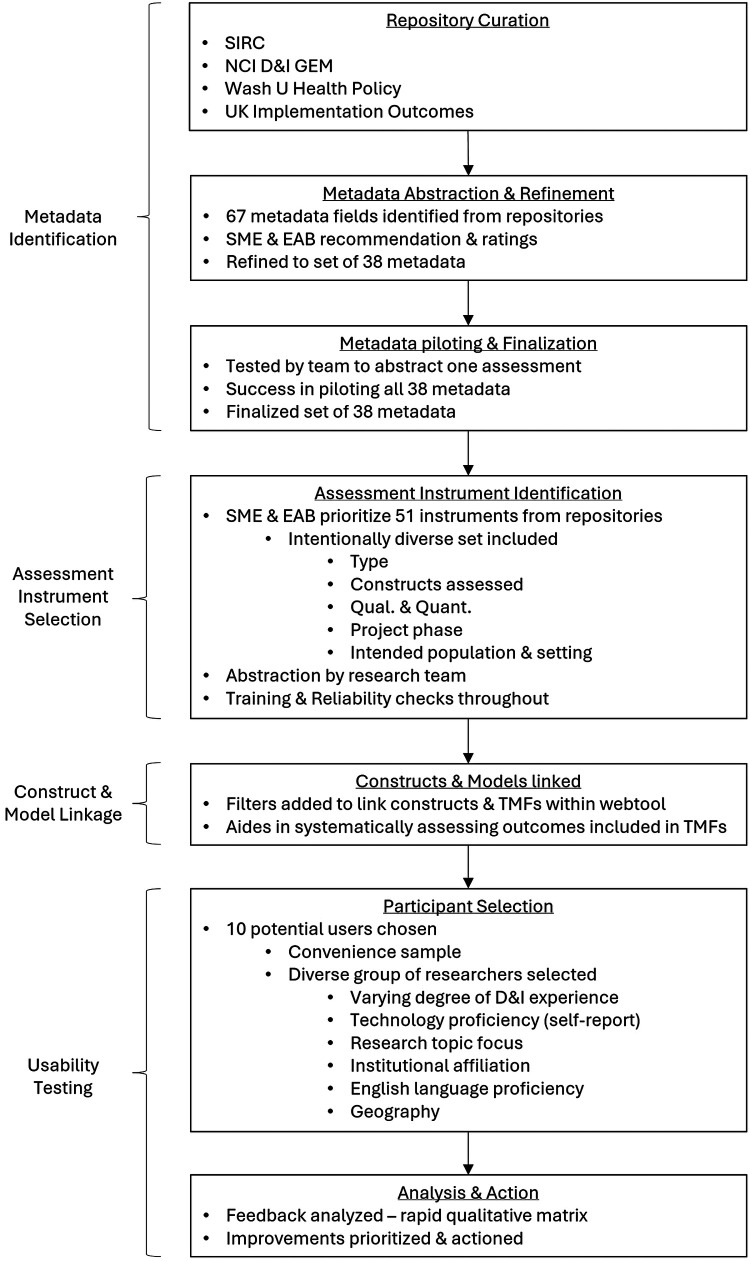
Metadata identification for assessment selection and operationalization.

To expand the Assess section of the D&I Models in Health Webtool, we followed a multistep, user-centered development (UCD) process that combined expert input, structured abstraction, iterative piloting, and usability testing. UCD is an iterative design approach that actively involves end users throughout the development process to ensure that the resulting product meets their needs, preferences, and workflow demands (16). UCD has been increasingly applied in implementation science and health informatics contexts to improve the usability, relevance, and uptake of digital tools and decision supports (17). In the context of this work, UCD involved a systematic engagement of diverse potential and actual users of the webtool across the planning, development, and release phases to inform design decisions and iteratively improve the content, functionality, and interface of the expanded Assess section and repository. This work was supported by the National Cancer Institute (NCI), the National Heart, Lung, and Blood Institute (NHLBI), and the Office of Disease Prevention (ODP) (NIH Supplement P50CA24468805S2). Figure 2 summarizes the process that we used for (a) metadata identification, (b) assessment instrument selection, (c) construct and model linkage, and (d) usability testing geared toward end user success.

### 2.2 Repository curation

We curated a set of existing, reputable repositories (Table 1) to provide an initial metadata list and a sampling frame for candidate assessment instruments:

- SIRC Instrument Review Project (IRP), which catalogs instruments mapped to Proctor's outcomes and CFIR constructs and documents PAPERS psychometric/pragmatic ratings (18).
- NCI GEM, including the historic GEM D&I initiative, which disseminated measures recommended through NIH/NCI efforts (19).
- Washington University Health Policy Measures repository, developed through a systematic review of policy implementation measures with PAPERS ratings (20).
- Implementation Outcome Repository (UK), a free, continuously updated resource led by King's College London and partners, focused on measurement of implementation outcomes (21).

**Table 1:** Existing assessment instrument repositories referenced.

Name of repository	Brief description	Link to repository
Society for Implementation Research Collaboration (SIRC) instrument repository	A member-access repository that systematically reviews and synthesizes implementation science measures, organized by Proctor's implementation outcomes and CFIR constructs; each measure was rated with the PAPERS scale for psychometric and pragmatic quality. Users can browse constructs to find linked instruments to summaries, sample items, scoring guides, related literature, and visual PAPERS profiles.	https://societyforimplementationresearchcollaboration.org/sirc-instrument-project/
The National Cancer Institute's Group-Evaluated Measures (GEM) (originally Grid-Enabled Measures)	GEM started as an open-source, online collaborative tool to support researchers and practitioners to identify, assess, and recommend self-reported measures across many domains of cancer-related research. A D&I measures working group included D&I science-relevant measures and related metadata (1).	Original version of GEM is no longer available. A revised and repurposed version is available at: https://cancercontrol.cancer.gov/brp/research/group-evaluated-measures
Washington University at Saint Louis's Health Policy Group instrument repository	A repository to help policy researchers, evaluators, and implementation science researchers identify and select measures to assess the implementation of health policies in a variety of settings (e.g., hospitals, outpatient clinics, neighborhoods, and schools) (22).	https://www.health-policy-measures.org/
Implementation Outcomes repository developed by the Centre for Implementation Science at King's College London, King's Improvement Science and the Behavioural and Implementation Science research group at the University of East Anglia	A free online resource for implementation stakeholders, including researchers and healthcare professionals, wishing to quantitatively measure implementation outcomes.	https://implementationoutcomerepository.org/

These sources provided a diverse universe of candidate instruments spanning constructs, settings, and methods (quantitative and qualitative) and ensured alignment with implementation outcomes (e.g., acceptability, adoption, appropriateness, feasibility, fidelity, penetration, cost, and sustainability) (Figure 2: top panel).

### 2.3 Expert engagement (Subject Matter Experts and Expert Advisory Board)

We convened a multidisciplinary Subject Matter Expert (SME) group (*n* = 3; frequent, deep engagement) and an Expert Advisory Board (EAB) (*n* = 9; periodic, higher-level consultants) to guide the full process of identifying/refining metadata, sharpening inclusion criteria, prioritizing assessments for abstraction, advising on usability testing, and informing sustainability planning. The SME and EAB were selected carefully to include colleagues with expertise in measurement development. These individuals were the originators of the repositories that we referenced (Table 1), specific methodological experts (qual/quant), and pioneers of novel measurement techniques. SME selection emphasized breadth spanning quantitative and qualitative instrument development/use, while the EAB spanned D&I measurement, psychometrics, and emergent methods.

### 2.4 Metadata characteristic identification

#### 2.4.1 Abstraction and refinement

From the curated repositories, the core research team abstracted 67 preliminary metadata fields, defined as standardized “characteristics of assessment instruments” (e.g., instrument purpose, outcome(s) measured, setting, population, administration mode, scoring, burden, language availability, and psychometric/pragmatic evidence). This list was iteratively reviewed and expanded with input from SMEs and the EAB and then refined to a candidate set of 38 metadata fields for piloting (Table 2).

**Table 2:** Metadata or key characteristics for assessment instruments in the Assess section of the webtool.

Metadata/characteristic	Description	Characteristic type	Answer options
General overview			
Brief description	A brief summary description of the assessment instrument	Open text	
Citing literature original	Reference for publications describing the development of the assessment instrument	Open text	
Citing literature - application	Reference for publications on the application of the assessment instrument	Open text	
Website	The website providing access to and/or describing the assessment instrument	Open text	
Version	Number/name of the most recent version of the assessment instrument	Open text	Version number Not found
Attachments	Related files uploaded (instrument if directly available) including descriptions for each	Attachment/upload	
Related instruments	Related files uploaded (instrument if directly available) including descriptions for each	Multiple choice	List of existing instruments
Summary characteristics			
Qualitative/quantitative	The assessment instrument uses quantitative and/or qualitative data	Multiple choice	Qualitative Quantitative Both
Type of instrument	The type of assessment instrument	Single choice	Survey Individual interview Focus group interview Periodic reflection Observation guide Documentation guide EMR form Other (specify)
Number of items	Number of items in the assessment instrument	Open text	Numerical (1–250)
Subscales	Names of each of the subscales and the number of items for each of the subscales	Open text	Name of subscale 1 (#of items) Name of subscale 2 (# of items) No subscales
Language	The language(s) in which the assessment instrument is available	Multiple choice	English Spanish Other (specify)
Implementation Science Considerations			
Constructs	Constructs assessed by the assessment instrument (linked to constructs included in the D&I models webtool)	Multiple choice	Acceptability/feasibility Adaptation and evolution Adopter/implementer/decision-maker characteristics Adoption Awareness Barriers and facilitators Champion/field agent Communication Communication channels Compatibility Complexity Context Context—Inner setting Context—Outer setting Cost Development of an intervention Dissemination Dose Engagement Evaluation External validity/generalizability Fidelity Fit Goals Health equity Identification Implementation Innovation characteristics Knowledge and Knowledge synthesis Knowledge transfer and utilization Maintenance and sustainability Observability Outcomes Outcomes—Health/QOL/Satisfaction/Clinical Outcomes—Implementation Outcomes—Quality improvement/practice or policy change Patient/target audience characteristics and needs Preimplementation Process Reach Readiness Relative advantage Stakeholders Strategies Translation Trialability
Theories, Models, and Frameworks (TMFs) relevant	The IS TMFs relevant for the assessment instrument based on the constructs assessed	Multiple choice	List of all TMFs relevant
Strategies	The implementation strategy (strategies) evaluated by the assessment instrument	Multiple choice	Use evaluative and iterative strategies Provide interactive assistance Adapt and tailor to context Develop stakeholder interrelationships Train and educate stakeholders Support clinicians Engage consumers Utilize financial strategies Change infrastructure
Implementation Science outcomes	The relevance of the assessment instrument to various implementation outcomes	Multiple choice	Acceptability Adaptation Adoption Appropriateness Effectiveness Feasibility Fidelity Implementation Implementation cost Penetration Reach Sustainment
Phase of the implementation process	The phase of the implementation process when the assessment instrument can be used	Multiple choice	Preimplementation Implementation Sustainment Not found
Intended Focus			
Levels of data collection	The level(s) from which the assessment instrument collects data	Multiple choice	Individual (patient, community member) Implementer Organizational Community System Policy
Intended priority population	The intended priority population from whom data are collected using the assessment instrument	Multiple choice or open text	Community members/Patients Researcher/Evaluator Clinician Administrator Public health practitioner Teacher/Trainer Employer Other (specify)
Intended priority setting	The intended priority setting in which the assessment instrument is used	Multiple choice or open text	Clinical Inpatient Residential care Community organization Public health agency School Workplace Other (specify)
Policy	The assessment instrument is relevant to policy	Single choice	Public (laws, regulations) Healthcare financing/Reimbursement Clinical practice Not found
Equity	The assessment instrument includes at least one equity-relevant component	Single choice	Yes Not found
Psychometric Properties			
Scoring	The assessment instrument produces a composite score	Single choice	Yes No Not found
Norms	Measures of central tendency and distribution for the total score are based on small, medium, and large sample sizes	Single choice	Yes Not found
Responsiveness	The ability of the assessment instrument to detect change over time	Single choice	Yes Not found
Validity	The extent to which an instrument measures what it is intended to measure accurately	Multiple choice	Concurrent validity (Definition) Convergent validity (Definition) Cross-cultural validity (Definition) Discriminant validity (Definition) Known group validity (Definition) Predictive validity (Definition) Unspecified validity
Reliability	The extent to which results are consistent over time, across raters, across settings, or across items intended to measure the same thing	Single choice	Yes Not found
Factor Analysis	A statistical method that uses the correlation between observed variables to identify common factors	Single choice	Yes Not found
Pragmatic Properties			
Time to administer	The amount of time required to complete the assessment instrument	Open text	Number of minutes “as reported”
Secondary data			
Cost	Cost associated with access to the assessment instrument (some instruments might require login)	Single choice	Free Cost Not found
Literacy	Readability of the items reported on	Single choice	Yes Not found
Interpretation	Expertise needed for interpretation of data is reported	Single choice	Yes No Not found
Training	Expertise needed to sue the assessment instrument is reported	Single choice	Yes No Not found
Resources required to administer	Resources needed to administer the assessment instrument (FTE for data collector, equipment etc.)	Single choice	None/low High Not found
User guidance	Guides are provided to support the administration of assessment instrument/data collection, and/or analysis of data from the assessment instrument, and/or interpretation of data, and/or action/decision on how to use data	Multiple choice	Guidance for Action/Decision Guidance to Administer Guidance to Analyze Guidance to Interpret
Obtrusiveness	Degree of intrusion the participants will experience because of data collection when using the assessment instrument (e.g., assessment instruments that rely on the use of secondary data or automated data will be less obtrusive)	Single choice	High: Observation and synchronous collection of data Low: Automated collection of data or secondary data Medium: Asynchronous collection of data
Interactivity	Data collection and/or result generation involves interactive components	Multiple choice	Adaptive instrument Generative outcome Not found

To promote harmonization with the field, we drew on widely used frameworks and guidance for implementation, measurement, and reporting (e.g., Proctor's outcomes taxonomy, CFIR, PRISM, and PAPERS criteria embedded in the repositories).

#### 2.4.2 Rating and decision rules

Each metadata field was independently rated by the core team on two 3-point scales: *usefulness* and *feasibility* (1 = Not; 2 = Somewhat; 3 = Very). Mean scores guided inclusion decisions using the following thresholds:

- <2.0 = exclude
- 2.0–2.5 = flag for discussion
- >2.5 = include

Usefulness was rated first, with BR and RGl adjudicating items flagged for discussion. We then repeated the process for feasibility to finalize the pilot list. All justifications and comments were documented. We refined metadata names/definitions and drafted preliminary rating rubrics to standardize how each characteristic should be abstracted.

#### 2.4.3 Piloting and finalization

To pilot the metadata fields, we selected three well-known instruments (one quantitative survey, two qualitative guides), informed by SME recommendations. Qualitative advisors suggested adding an interview/focus group guide and an observation guide to ensure representation of common qualitative characteristics. Following SME and EAB review, we conducted pilot abstraction and confirmed the team’s ability to consistently code all 38 fields. These 38 metadata were then finalized (Table 2) for use in full abstraction.

### 2.5 Assessment instrument selection

#### 2.5.1 Prioritization and sampling

Using the finalized metadata, SMEs and the EAB jointly prioritized 51 high-priority instruments from the four repositories (Table 1). We intentionally sampled for diversity on the following:

- Instrument type (e.g., scales, checklists, interview/focus group guides, and observation tools)
- Constructs assessed (spanning implementation outcomes and determinants)
- Method (quantitative and qualitative)
- Implementation phase, population, setting, and equity relevance

### 2.6 Abstraction procedures and reliability

A trained abstraction team completed structured metadata abstraction for each instrument using a standardized codebook and practice cases. We embedded training sessions and reliability checks throughout (calibration meetings, discrepancy logs, consensus rules). The process was designed to be replicable to enable future rounds of instrument addition without sacrificing consistency.

### 2.7 Construct and model linkage

For each instrument, we mapped measured constructs to IS theories, models, and frameworks (TMFs) included in the webtool. We implemented filters to allow users to search by construct and TMF, see which instruments operationalize a given construct, and view measurement coverage for constructs emphasized by a TMF (e.g., CFIR constructs and Proctor outcomes). This linkage operationalizes long-standing guidance encouraging explicit ties between TMFs and measurement, addressing a frequent gap noted in the literature and grant reviews.

### 2.8 Usability testing

We recruited a convenience sample of 10 potential users, intentionally capturing diversity in IS experience, technology proficiency (self-reported), research topic, institutional affiliation, English language proficiency, and geography. These participants were chosen through personal connections and close colleague recommendations where gaps existed. Participants completed a brief orientation and then performed task-based walkthroughs of the new Assess section features using a think-aloud protocol. Sessions were screen-recorded with field notes. We elicited reflections on clarity, navigation, and decision support (e.g., how easily participants could locate instruments, interpret metadata, and use filters to connect constructs to TMFs).

### 2.9 Analysis and iteration

We synthesized feedback using a rapid matrix-based qualitative analysis, collating issues and improvement opportunities by page/feature and by metadata element. The team prioritized and implemented changes in short cycles (content wording, visual cues, definitions, tutorials), consistent with prior human-centered refinements to the D&I Models Webtool.

### 2.10 Governance and sustainability

The expanded Assess section, including metadata definitions, abstraction codebook, and linkage filters, is maintained via the webtool’s established governance processes, enabling periodic instrument additions and updates to preserve consistency and relevance over time.

## 3 Results

### 3.1 Overview of coded metadata characteristics

A total of 38 assessment instrument characteristics (i.e., metadata fields) were identified through our multistep rating process with the advisors. We organized these under six key domains. A detailed description of each characteristic and its assessment is provided in Table 2.

### 3.2 Description of included assessment instruments

A total of 51 assessment instruments were included in Phase 1 of the tool, selected based on iterative input from our content experts and advisors. Table 3 provides examples of the basic characteristics of these assessment instruments, with the full list available in Supplementary Material 1. In our purposeful sample, we aimed to include a diverse range of instruments in terms of their type (e.g., survey and interview guide), the implementation science (IS) constructs that they assess (e.g., strategies, health equity, and context), data format (quantitative vs. qualitative), implementation phase (e.g., implementation and sustainment), and intended priority target audiences and settings (e.g., clinical and public health).

**Table 3:** Example list of assessment instruments (full list available in Supplementary Material 1 and on the webtool with metadata: https://dissemination-implementation.org/tool/assess/).

Name of instrument	Citing literature—development/original	Type of instrument	Constructs assessed	Phase of implementation process
Framework for documenting modifications to implementation strategies in healthcare (FRAME-IS) adaptation tracking instrument	Miller CJ, Barnett ML, Baumann AA, Gutner CA, Wiltsey-Stirman S. The FRAME-IS: a framework for documenting modifications to implementation strategies in healthcare. Implement Sci. 2021;16 (1):36. doi:10.1186/s13012-021-01105-3	Survey	Strategies	Implementation
Goodman's Level of Institutionalization	Goodman RM, Steckler A. A framework for assessing program institutionalization. Knowl Soc. 1989;2 (1):57–71. Goodman RM, McLeroy KR, Steckler AB, Hoyle RH. Development of level of institutionalization scales for health promotion programs. *Health Educ Q*. 1993;20 (2):161–178. doi:10.1177/109019819302000208	Survey	Adoption	Sustainment
AHRQ Digital health equity framework	Agency for Healthcare Research and Quality. Evidence- and consensus-based digital healthcare equity framework. AHRQ Publication No. 24-0020-1-EF. Published February 2024.	Other (Checklist)	Adopter/Implementer/Decision-maker characteristics Context—Outer setting Context—Inner setting Health equity Patient/Target audience characteristics and needs Outcomes Outcomes—Practice or policy change	PreImplementation Implementation Sustainment
Research Engagement Survey Tool - 9 item	Goodman MS, Ackermann N, Pierce KA, Bowen DJ, Thompson VS. Development and validation of a brief version of the research engagement survey tool. Int J Environ Res Public Health. 2021;18 (19):10020. doi:10.3390/ijerph181910020	Survey	Engagement Health equity Stakeholder	PreImplementation Implementation Sustainment
CFIR Interview Guide Webtool	Original: Damschroder LJ, Aron DC, Keith RE, Kirsh SR, Alexander JA, Lowery JC. Fostering implementation of health services research findings into practice: a consolidated framework for advancing implementation science. Implement Sci. 2009;4:50. doi:10.1186/1748-5908-4-50 Updated: Damschroder LJ, Reardon CM, Widerquist MAO, Lowery J. The updated Consolidated Framework for Implementation Research based on user feedback. Implement Sci. 2022;17 (1):75. doi:10.1186/s13012-022-01245-0	Individual interview Focus group interview	Adaptation and evolution Adopter/Implementer/Decision-maker characteristics Barriers and facilitators Compatibility Context—Inner setting Context—Outer setting Cost Engagement Evaluation Innovation characteristics Outcomes Process Readiness Relative advantage Stakeholder Strategies	PreImplementation Implementation

**Figure 3:**
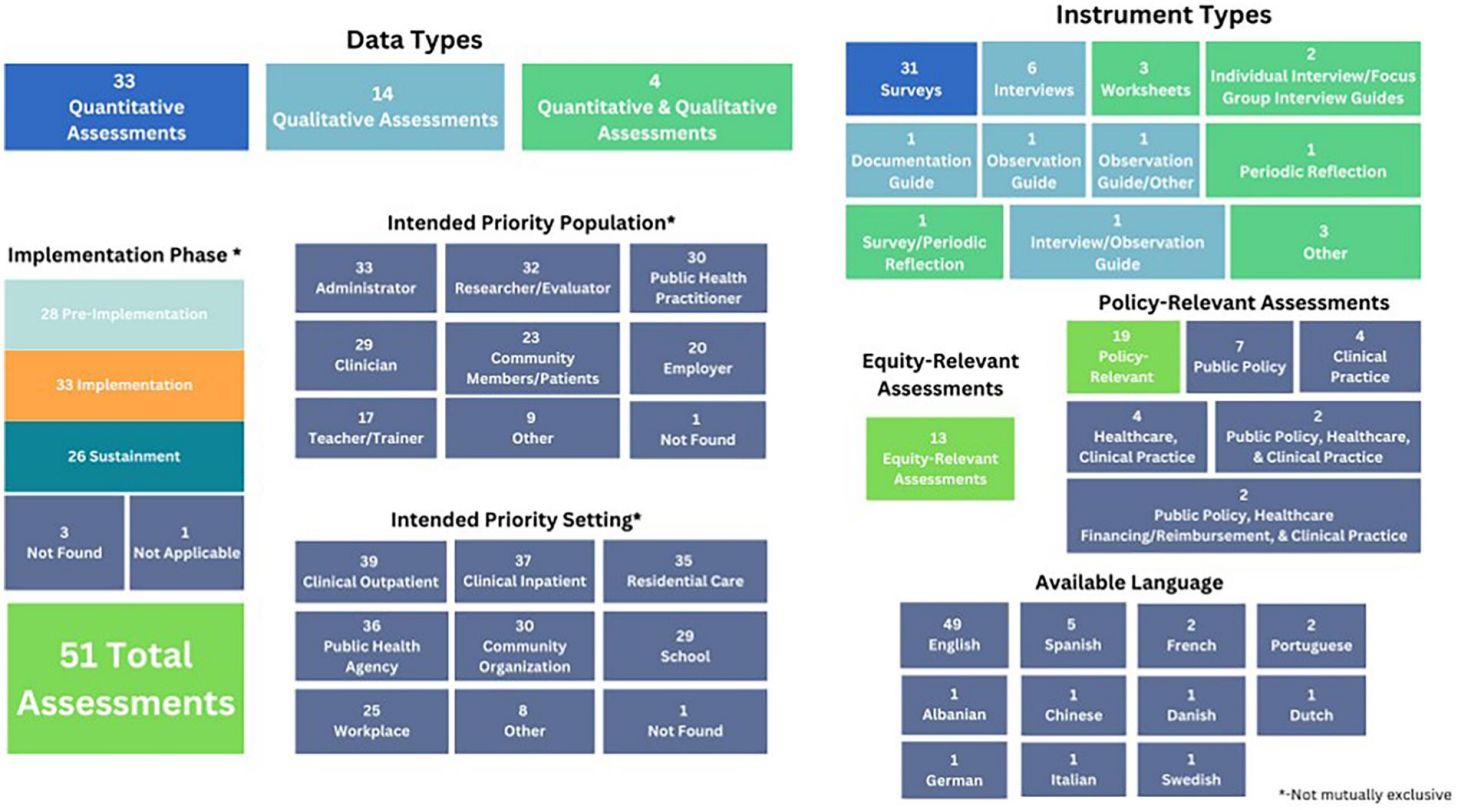
Descriptive characteristics of included assessment instruments.

As shown in Figure 3, the instruments included 33 quantitative assessments, 14 qualitative assessments, and four mixed assessments that incorporated both quantitative and qualitative data. The most common instrument types were surveys (*n* = 31), followed by interviews (*n* = 6), worksheets (*n* = 3), and other formats such as observation guides and periodic reflections. Within the broader categories for instrument type, there was a mix of data types. For example, within the “worksheets” category, two instruments were considered quantitative, and one was considered qualitative. The instruments were designed for various implementation phases, with 28 targeting preimplementation, 33 addressing implementation, and 26 focusing on sustainment.

The assessments were intended for a range of priority audiences (many for multiple target audiences), including administrators (*n* = 33), researchers/evaluators (*n* = 32), public health practitioners (*n* = 30), and clinicians (*n* = 29). In addition, the instruments covered diverse priority settings such as clinical outpatient (*n* = 39), clinical inpatient (*n* = 37), and public health agencies (*n* = 36).

Furthermore, the tool includes 13 equity-relevant assessments and 19 policy-relevant instruments, with subcategories addressing public policy (*n* = 7) and clinical practice (*n* = 4). The majority of assessments are available in English (*n* = 49), with some in Spanish (*n* = 5), French (*n* = 2), and other languages.

The information provided on the 38 metadata includes the presence or absence of data on the characteristics of interest (e.g., test–retest reliability). For multiple reasons, including differences in cross-content areas, experimental procedures, and specific analytic approaches, we do not provide specific values (e.g., *r* = 0.7) or judgments about whether this is a good or poor value. Moreover, the information included is in reference to the seminal article(s) on the instrument. We have not conducted a systematic review to assess all possible studies using a given instrument. Such an assessment is beyond the scope of this tool and would need continual updating.

### 3.3 Functionalities and integration of the Assess section of the webtool

To enhance the functionality of the Assess section and ensure seamless integration with the broader webtool, multiple features were developed and refined. Upon entering the Assess section, site visitors can engage with the tool in various ways, including accessing guidance resources such as a video tutorial, exploring available instruments, and reviewing key characteristics of the instruments upon selecting and using IS assessment instruments. These elements were designed to support users regardless of their entry point, prior knowledge, or specific purpose for visiting the site, facilitating the practical application of the instruments in their work.

Guidance for selecting and applying IS assessment instruments was developed in collaboration with the Expert Advisory Board, which provided content expertise to assist visitors in effectively using the tool. This guidance is presented on a stand-alone page and is organized into two categories: selecting and using IS assessment instruments, and additional information on the Assess section. The first category provides instructions on interacting with the tool, explains key characteristics of the assessment instruments, and offers insights on how to apply them—particularly qualitative instruments. The second category provides additional considerations for selecting instruments, clarifies that inclusion in the tool does not imply endorsement or recommendation, and describes how constructs were selected and organized within the webtool.

To support different user needs, the webtool allows visitors to explore IS assessment instruments through multiple pathways. Users can browse by instrument to view individual assessment tools, by IS construct to identify instruments aligned with specific implementation science constructs, or by IS model to locate instruments associated with established IS frameworks. These options ensure that visitors can navigate the Assess section based on their project needs, existing knowledge, and search preference, enhancing the usability of the webtool.

To further optimize functionality, filters were implemented to refine search results based on a subset of key characteristics of the instruments (a full list of key characteristics is provided in Table 2). These characteristics include instrument type, data format (qualitative or quantitative), language, phase of the implementation process, constructs assessed, implementation outcomes, and equity relevance. In addition to the filtering system, a search bar was integrated to enable users to locate specific instruments efficiently. The search function was designed to recognize multiple variations of an instrument's name, including abbreviations, acronyms, and alternative word combinations, ensuring comprehensive search functionality and ease of access.

Each IS assessment instrument is linked to relevant IS theories, models, and frameworks (TMFs), as well as other related instruments. This interconnectivity extends the usability of the Assess section beyond a stand-alone feature, supporting the broader goal of operationalizing implementation activities across the webtool. By establishing these links, the webtool allows users to explore how different instruments relate to theoretical models, constructs, and other components of implementation science.

### 3.4 Usability findings and refinements

As part of the refinement process, we reviewed and addressed a total of 187 usability recommendations from 10 testers and EAB members, of which 113 were unique. Following a systematic evaluation, we implemented changes for 74 recommendations within the current round of funding, while an additional 20 were identified for future implementation pending further funding. To ensure a structured and methodical approach to usability refinement, the team systematically reviewed each suggestion, reported errors, requested change, or identified an area of confusion through usability testing. Each issue was assessed to determine an appropriate solution, followed by a decision-making process regarding the timing and feasibility of implementing the change. Examples of recommendations include fixing broken links, alignment of terminology used, font color changes, and additional disclaimer text when linking to external sites that we do not manage.

Prioritization of changes was based on several key factors, including the type of issue, the urgency of the modification, and the resources required for implementation. Changes that could be addressed directly by the central team, such as modifications to content, terminology, and minor interface adjustments, were implemented internally. More complex refinements, particularly those requiring technical modifications to functionality or navigation, were escalated to the web development team. This tiered approach ensured that high-priority usability issues were resolved efficiently, while aligning with available resources and funding constraints.

The changes made were categorized into five thematic areas: terminology and labeling; user interface and navigation; functionality and features; content and information; and accessibility and usability. Refinements related to terminology and labeling focused on improving clarity in wording, labels, and metadata descriptions, addressing issues such as confusing labels and inaccurate metadata terms. Modifications in user interface and navigation were aimed at enhancing user interaction and site navigation by restructuring menus, adjusting button placements, and refining page layouts to streamline usability. Changes categorized under functionality and features involved enhancing core functionalities, including the addition of new filters, interactive components, and tool-based improvements. Content and information updates primarily addressed missing or unclear descriptions, enhanced instructional content, and the improved guidance to support user comprehension. Last, accessibility and usability refinements focused on improving visual design, readability, and overall user-friendliness, including adjustments to font size and color contrast.

**Figure 4:**
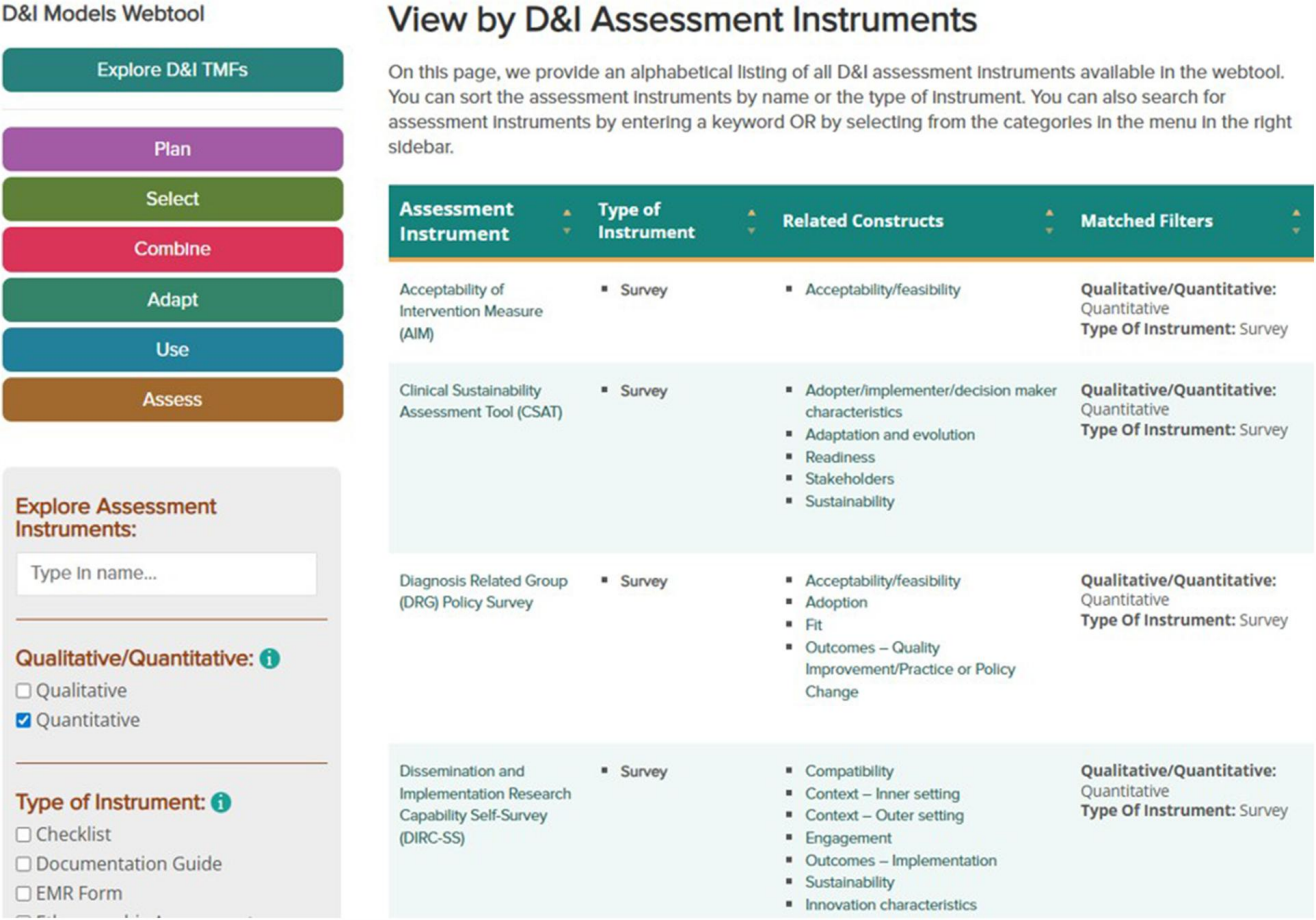
Assess search functions and auto-refreshed result output.

Among the 74 recommendations addressed in the current funding cycle, the largest proportion of changes focused on content and information (*n* = 33; 35%) and user interface and navigation (*n* = 32; 34%), reflecting a strong emphasis on enhancing the clarity of instructional content and optimizing site navigation. Changes related to terminology and labeling (*n* = 16; 17%) aimed to improve consistency and reduce confusion in the labeling of key elements within the tool. In addition, functionality and features (*n* = 11; 12%) updates contributed to improved interactivity and tool usability, while a smaller number of refinements focused on accessibility and usability (*n* = 2; 2%), addressing readability and visual design concerns. The final section of this interactive tool is shown in Figure 4.

The remaining 20 usability recommendations have been earmarked for future refinement, contingent upon additional funding. These pending changes primarily focus on expanding functionality, refining accessibility features, and further enhancing content presentation to improve user experience.

## 4 Discussion

We expanded the D&I Models Webtool by redesigning and substantially expanding the Assess section, creating a publicly available, construct-linked tool of implementation assessment instruments. The Assess section uniquely connects IS TMFs and constructs to specific instruments across quantitative, qualitative, and mixed-methods modalities, accompanied by a transparent metadata schema (38 fields) to support search, comparison, and selection of assessments. This collection represents a broad spectrum of implementation-relevant assessments to support diverse stakeholders across various implementation phases and settings. In Phase 1, the tool includes 51 instruments spanning multiple phases (preimplementation, implementation, sustainment), settings (clinical outpatient and inpatient, public health), priority users (administrators, clinicians, public health practitioners, researchers), and topical emphases such as equity-relevant and policy-relevant assessments.

The expansion of the Assess section of the D&I Models Webtool addresses a critical gap in implementation science: the lack of accessible, curated, and construct-linked assessment instruments, ultimately facilitating a more integrated and systematic approach to implementation research. To our knowledge, this is the only publicly available webtool that systematically connects IS assessment instruments to TMFs and constructs, supporting researchers and practitioners in selecting context-appropriate tools. Although other websites specific to a given TMF (e.g., EPIS, CFIR, and RE-AIM) provide information on assessments relative to that TMF, we suggest that it is also the only interactive resource designed to help users search for and identify assessments across different TMFs.

Our development process emphasized user-centered design, expert engagement, and iterative refinement. The inclusion of diverse assessment methods, beyond surveys, responds to the need of the field for more varied and context-sensitive assessment instruments. The metadata structure enhances usability by allowing users to filter and search instruments based on several key characteristics such as implementation phase, data format, equity relevance, and intended population.

Through this systematic decision-making process, the team effectively addressed usability concerns, ensuring that modifications were both strategically implemented and aligned with the overarching goals of improving the usability, integration, and overall user experience of the Assess section.

Earlier IS measurement repositories have been valuable but have faced access, scope, or longevity constraints. The Assess section contributes a free, public, construct-first alternative that integrates directly into the D&I Models Webtool (Plan, Select, Combine, Adapt, Use, Assess). Organizing by TMF construct and providing crosswalk models support theory-driven measurement choices in ways that disease- or setting-only catalogs often cannot.

### 4.1 Limitations

Despite its strengths, the Assess section has limitations. This Phase 1 collection is purposeful rather than exhaustive, and some domains (e.g., policy and system-level constructs, structural determinants, and non-English instruments) remain underrepresented. Evidence quality is heterogeneous across instruments; importantly, *inclusion indicates relevance and availability of information and not quality or endorsement*. Finally, maintenance and expansion are resource-dependent. At the launch of the Assess section, we included 51 instruments. We are continuing to add assessments and prioritize those we think will be of the broadest relevance. We invite users to suggest additional assessments and provide feedback, as this is a continually evolving product. Users can currently do this through the “Submit TMFs” link in the toolbar on the webtool. Our team reviews these submissions on a quarterly basis and abstracts or adds TMFs/assessments that meet our inclusion criteria. Because of limited capacity, we will not be able to notify users after the request is processed. We plan to make this more explicit by collecting assessments in a manner similar to TMFs via a separate link and form.

### 4.2 Future directions

Future directions include adding brief instructional videos, expanding the instrument database, enhancing guidance for instrument selection, and incorporating crowd-sourced feedback. The Assess section also offers a foundation for identifying gaps in available instruments, guiding future development in the field of IS. By linking assessment instruments to TMFs and constructs, the Assess section supports more rigorous, theory-informed implementation research and practice, helping users operationalize IS activities across diverse settings. Since the release of this instrument, we have added instruments and guidance to improve the Assess section, although this article reports only the initial phase. By addressing usability concerns systematically, the refinements contribute to a more accessible, user-friendly, and effective Assess section within the webtool. As time and resources permit, we will continue to add new instruments and welcome recommendations.

### 4.3 Conclusions

Linking IS TMFs to usable, context-appropriate assessment instruments is the most direct route from theory to practice in implementation research. By delivering a public, curated, construct-linked tool with transparent metadata, the Assess section provides foundational infrastructure for more rigorous, comparable, and pragmatic IS studies across diverse settings. Continued research, community input, and sustained support will be essential to expand coverage and deepen the impact of this resource on the quality and comparability of implementation science.

## Data Availability

The datasets presented in this study can be found in online repositories. The names of the repository/repositories and accession number(s) can be found at: https://dissemination-implementation.org/tool/assess/.
