# Supplementary material for: Implementation research assessment instruments: a webtool to identify, compare and select assessment instruments connected with implementation science theories, models, and frameworks
Source: Front Health Serv. 2026 Jul 24;6:1855885. doi: 10.3389/frhs.2026.1855885 (PMC13447451; doi:10.3389/frhs.2026.1855885)
Supplement: Supplementary file 1 [file Table1.docx]

| **Supplementary Materials 1. Full list of assessment instruments (full metadata available on the webtool: https://dissemination-implementation.org/tool/assess/)** | | | | |
| --- | --- | --- | --- | --- |
| Name of instrument | Citing Literature - Development/original | Type of Instrument | Constructs Assessed | Phase of Implementation Process |
| Iterative, Practical, Robust Implementation and Sustainability Model (iPRISM) webtool | Trinkley, K.E., Glasgow, R.E., D’Mello, S. et al. The iPRISM webtool: an interactive tool to pragmatically guide the iterative use of the Practical, Robust Implementation and Sustainability Model in public health and clinical settings. Implement Sci Commun 4, 116 (2023). <https://doi.org/10.1186/s43058-023-00494-4> | Survey   Periodic reflection | Acceptability/feasibility   Adaptation and evolution   Adopter/Implementer/Decision maker characteristics   Barriers and facilitators   Communication   Complexity   Context – Inner setting   Cost   Health Equity   Innovation characteristics   Knowledge and knowledge synthesis   Sustainability   Outcomes – Health/QOL/Satisfaction/Clinical   Outcomes – Quality improvement/Practice or policy change   Patient/target audience characteristics and needs   Readiness   Stakeholders | Pre-Implementation   Implementation   Sustainment |
| Feasibility of Intervention measure (FIM) | Weiner BJ, Lewis CC, Stanick C, Powell BJ, Dorsey CN, Clary AS, Boynton MH, Halko H. Psychometric assessment of three newly developed implementation outcome measures. Implement Sci. 2017 Aug 29;12(1):108. doi: 10.1186/s13012-017-0635-3. PMID: 28851459; PMCID: PMC5576104. | Survey | Feasibility | Pre-Implementation |
| Cost of Implementing New Strategies (COINS) | Saldana L, Chamberlain P, Bradford WD, Campbell M, Landsverk J. The Cost of Implementing New Strategies (COINS): A Method for Mapping Implementation Resources Using the Stages of Implementation Completion. Child Youth Serv Rev. 2014 Apr 1;39:177-182. doi: 10.1016/j.childyouth.2013.10.006. PMID: 24729650; PMCID: PMC3979632. | Template/Worksheet | Cost | Pre-Implementation   Implementation   Sustainment |
| Implementation Climate Scale (ICS) | Ehrhart, M.G., Aarons, G.A. & Farahnak, L.R. Assessing the organizational context for EBP implementation: the development and validity testing of the Implementation Climate Scale (ICS). Implementation Sci 9, 157 (2014). <https://doi.org/10.1186/s13012-014-0157-1>    Ehrhart MG, Torres EM, Hwang J, Sklar M, Aarons GA. Validation of the Implementation Climate Scale (ICS) in substance use disorder treatment organizations. Subst Abuse Treat Prev Policy. 2019 Aug 23;14(1):35. doi: 10.1186/s13011-019-0222-5. PMID: 31443666; PMCID: PMC6708170. | Template/Worksheet | Adaptation and evolution   Complexity   Context - Inner setting   Evaluation   Innovation characteristics   Outcomes - Quality improvement/practice or policy change   Strategies | Pre-Implementation   Implementation   Sustainment |
| Framework for documenting modifications to implementation strategies in healthcare (FRAME-IS) adaptation tracking instrument | Miller CJ, Barnett ML, Baumann AA, Gutner CA, Wiltsey-Stirman S. The FRAME-IS: a framework for documenting modifications to implementation strategies in healthcare. Implement Sci. 2021 Apr 7;16(1):36. doi: 10.1186/s13012-021-01105-3. PMID: 33827716; PMCID: PMC8024675. | Survey | Strategies | Implementation |
| Longitudinal Implementation Strategy Tracking System (LISTS) | Smith JD, Norton WE, Mitchell SA, Cronin C, Hassett MJ, Ridgeway JL, Garcia SF, Osarogiagbon RU, Dizon DS, Austin JD, Battestilli W, Richardson JE, Tesch NK, Cella D, Cheville AL, DiMartino LD; IMPACT Consortium. The Longitudinal Implementation Strategy Tracking System (LISTS): feasibility, usability, and pilot testing of a novel method. Implement Sci Commun. 2023 Nov 28;4(1):153. doi: 10.1186/s43058-023-00529-w. PMID: 38017582; PMCID: PMC10683230. | Survey | Adaptation and evolution   Dose   Outcomes – Implementation | Implementation |
| Program Sustainability Assessment Tool (PSAT) | Luke DA, Calhoun A, Robichaux CB, Elliott MB, Moreland-Russell S. The Program Sustainability Assessment Tool: a new instrument for public health programs. Prev Chronic Dis. 2014 Jan 23;11:130184. doi: 10.5888/pcd11.130184. PMID: 24456645; PMCID: PMC3900326. | Survey | Adaptation and evolution   Communication   Context   Context – Inner setting   Context – Outer setting  Cost   Dissemination   Engagement   Evaluation | Sustainment |
| RAPICE Protocol, Activity, and Interview Prompt Guide | Palinkas LA, Zatzick D. Rapid Assessment Procedure Informed Clinical Ethnography (RAPICE) in Pragmatic Clinical Trials of Mental Health Services Implementation: Methods and Applied Case Study. Adm Policy Ment Health. 2019 Mar;46(2):255-270. doi: 10.1007/s10488-018-0909-3. PMID: 30488143; PMCID: PMC7427407. | Individual interview   Observation guide | Barriers and facilitators   Champion/Field agent  Communication   Communication channels   Compatibility   Complexity  Context   Context – Inner setting   Context – Outer setting   Cost   Dissemination   Fidelity   Evaluation   Health equity   Innovation characteristics Patient/target audience characteristics and needs   Reach   Stakeholders | Pre-Implementation   Implementation   Sustainment |
| RAPICE Summary Template | Palinkas LA, Zatzick D. Rapid Assessment Procedure Informed Clinical Ethnography (RAPICE) in Pragmatic Clinical Trials of Mental Health Services Implementation: Methods and Applied Case Study. Adm Policy Ment Health. 2019 Mar;46(2):255-270. doi: 10.1007/s10488-018-0909-3. PMID: 30488143; PMCID: PMC7427407. | Worksheet | Barriers and facilitators   Champion/Field agent   Communication   Communication channels   Compatibility  Complexity   Context   Context – Inner setting   Context – Outer setting   Cost   Dissemination   Fidelity   Evaluation   Health equity   Innovation characteristics   Patient/target audience characteristics and needs   Reach   Stakeholders | Pre-Implementation   Implementation   Sustainment |
| Readiness Thinking Tool - Observation Guide | Using readiness. Wandersman Center. (n.d.). <https://www.wandersmancenter.org/using-readiness.html> | Observation guide | Acceptability/feasibility   Adoption   Barriers and facilitators   Champion/Field agent   Communication   Communication channels   Compatibility   Complexity   Context   Context – Inner setting   Context – Outer setting   Cost   Implementation   Innovation characteristics   Knowledge and knowledge synthesis   Observability   Pre-implementation   Readiness | Pre-Implementation |
| Intervention Scalability Assessment Tool (ISAT) | Milat A, Lee K, Conte K, Grunseit A, Wolfenden L, van Nassau F, Orr N, Sreeram P, Bauman A. Intervention Scalability Assessment Tool: A decision support tool for health policy makers and implementers. Health Res Policy Syst. 2020 Jan 3;18(1):1. doi: 10.1186/s12961-019-0494-2. PMID: 31900230; PMCID: PMC6942323. | Survey | Adaptation and evolution Adopter/Implementer/Decision maker characteristics   Adoption   Context   Context – Inner setting   Context – Outer setting   Cost   Fidelity   Implementation   Sustainability  Reach   Readiness | Pre-Implementation   Implementation |
| Acceptability of Intervention measure (AIM) | Weiner, B.J., Lewis, C.C., Stanick, C. *et al.* Psychometric assessment of three newly developed implementation outcome measures. *Implementation Sci* **12**, 108 (2017). <https://doi.org/10.1186/s13012-017-0635-3> | Survey | Acceptability/Feasibility | Implementation   Sustainment |
| Appropriateness of Intervention measure (IAM) | Weiner, B.J., Lewis, C.C., Stanick, C. *et al.* Psychometric assessment of three newly developed implementation outcome measures. *Implementation Sci* **12**, 108 (2017). <https://doi.org/10.1186/s13012-017-0635-4> | Survey | Acceptability/Feasibility | Implementation |
| Clinical Sustainability Assessment Tool (CSAT) | Malone S, Prewitt K, Hackett R, Lin JC, McKay V, Walsh-Bailey C, Luke DA. The Clinical Sustainability Assessment Tool: measuring organizational capacity to promote sustainability in healthcare. Implement Sci Commun. 2021 Jul 17;2(1):77. doi: 10.1186/s43058-021-00181-2. PMID: 34274004; PMCID: PMC8285819. | Survey | Adopter/Implementer/Decision maker characteristics  Adaptation and evolution   Readiness   Stakeholders   Sustainability | Sustainment |
| Evidence Based Practice Attitude scale | Aarons GA. Mental health provider attitudes toward adoption of evidence-based practice: the Evidence-Based Practice Attitude Scale (EBPAS). Ment Health Serv Res. 2004 Jun;6(2):61-74. doi: 10.1023/b:mhsr.0000024351.12294.65. PMID: 15224451; PMCID: PMC1564126. | Survey | Acceptability/Feasibility   Adoption   Awareness   Readiness | Pre-Implementation   Implementation   Sustainment |
| Glisson's Organizational Social Context (OSC) | Glisson C, Landsverk J, Schoenwald S, Kelleher K, Hoagwood KE, Mayberg S, Green P; Research Network on Youth Mental Health. Assessing the organizational social context (OSC) of mental health services: implications for research and practice. Adm Policy Ment Health. 2008 Mar;35(1-2):98-113. doi: 10.1007/s10488-007-0148-5. Epub 2007 Dec 18. PMID: 18085434. | Survey | Context-Inner setting   Engagement   Readiness | Implementation   Sustainment |
| Van Schaik's Technology acceptance | Van Schaik, P., Bettany-Saltikov, J. A., & Warren, J. G. (2002). Clinical acceptance of a low-cost portable system for postural assessment. *Behaviour & Information Technology*, *21*(1), 47–57. <https://doi.org/10.1080/01449290110107236> | Survey | Acceptability/Feasibility   Adoption Adopter/Implementer/Decision maker characteristics   Compatibility | Implementation   Sustainment |
| Hall's Levels of Use Scale | Hall, G. E., Dirksen, D. J., & George, A. A. (2013). Measuring implementation in schools: Levels of use. Southwest Educational Development Laboratory. Kim, C., Kim, M. K., Lee, C., Spector, J. M., & DeMeester, K. (2013). Teacher beliefs and technology integration. Teaching and Teacher Education, 29, 76–85. <https://doi.org/10.1016/j.tate.2012.08.005> | Individual interview | Adoption   Awareness   Champion/Field agent   Identification | Pre-Implementation   Implementation   Sustainment |
| Landry's Knowledge Utilization Scale among Policymakers | Landry, Réjean, Nabil Amara, and Moktar Lamari. (2001). Climbing the Ladder of Research Utilization: Evidence from Social Science Research. Science Communication, 22(4), 396-422. <https://doi.org/10.1177/1075547001022004003> | Periodic reflection | Barriers and facilitators   Knowledege and knowledge synthesis   Knowledge transfer and utlization | Not applicable |
| Goodman's Level of Institutionalization | Goodman, Robert M., and Allan Steckler. "A framework for assessing program institutionalization." Knowledge in Society 2.1 (1989): 57-71. Goodman RM, McLeroy KR, Steckler AB, Hoyle RH. Development of level of institutionalization scales for health promotion programs. Health Educ Q. 1993;20(2):161-178. doi:10.1177/109019819302000208 | Survey | Adoption | Sustainment |
| Implementation Leadership Scale | Aarons, G.A., Ehrhart, M.G. & Farahnak, L.R. The implementation leadership scale (ILS): development of a brief measure of unit level implementation leadership. *Implementation Sci* **9**, 45 (2014). <https://doi.org/10.1186/1748-5908-9-45> | Survey | Champion/Field agent   Context - Inner setting   Knowledge and knowledge synthesis   Stakeholders | Not found |
| Normalization process theory questionnaire (NoMAD) | Finch TL, Girling M, May CR, et al. Improving the normalization of complex interventions: part 2 - validation of the NoMAD instrument for assessing implementation work based on normalization process theory (NPT). BMC Med Res Methodol. 2018;18(1):135. Published 2018 Nov 15. doi:10.1186/s12874-018-0591-x | Survey | Acceptability/Feasibility   Adoption  Awareness   Context - Inner setting   Engagement   Strategies | Implementation |
| AHRQ Digital health equity framework | Evidence- and Consensus-Based Digital Healthcare Equity Framework. (Prepared by Johns Hopkins University under Contract No. 75Q80120D00015.) AHRQ Publication No. 24-0020-1-EF. Rockville, MD: Agency for Healthcare Research and Quality. February 2024. | Other (Checklist) | Adopter/Implementer/Decision maker characteristics   Context - Outer setting   Context - Inner setting   Health equity  Patient/Target audience characteristics and needs   Outcomes   Outcomes - Practice or policy change | Pre-Implementation   Implementation   Sustainment |
| Stages of Implementation Completion | Chamberlain, Patricia, C. Hendricks Brown, and Lisa Saldana. "Observational measure of implementation progress in community based settings: the stages of implementation completion (SIC)." Implementation Science 6 (2011): 1-8. | Other (Undisclosed online tool) | Engagement   Acceptability/Feasibility   Readiness   Context - Inner setting   Stakeholders   Fidelity   Strategies   Evaluation   Sustainability | Pre-Implementation   Implementation   Sustainment |
| ORIC (Organizational Readiness for Implementing Change) | Shea, Christopher M., et al. "Organizational readiness for implementing change: a psychometric assessment of a new measure." Implementation science 9 (2014): 1-15. | Survey | Acceptability/Feasiblity   Readiness | Pre-Implementation |
| Research Engagement Survey Tool | Goodman MS, Ackermann N, Bowen DJ, Thompson V. Content validation of aquantitative stakeholder engagement measure.J Community Psychol. 2019;47:1937–1951. <https://doi.org/10.1002/jcop.22239> | Survey | Engagement   Health equity   Stakeholder | Pre-Implementation   Implementation   Sustainment |
| User Engagement Scale - Long | O'Brien, H.L. and Toms, E.G. (2010), The development and evaluation of a survey to measure user engagement. J. Am. Soc. Inf. Sci., 61: 50-69. <https://doi.org/10.1002/asi.21229>    O’Brien, Heather L., et al. “A Practical Approach to Measuring User Engagement with the Refined User Engagement Scale (UES) and New UES Short Form.” International Journal of Human-Computer Studies, vol. 112, Apr. 2018, pp. 28–39, <https://doi.org/10.1016/j.ijhcs.2018.01.00> | Survey | Engagement | Not found |
| Normalization processing theory interview guide | Thomas, L. H., French, B., Sutton, C. J., Forshaw, D., Leathley, M. J., Burton, C. R., Roe, B., Cheater, F. M., Booth, J., McColl, E., Carter, B., Walker, A., Brittain, K., Whiteley, G., Rodgers, H., Barrett, J., Watkins, C. L., & on behalf of the ICONS project team and the ICONS patient, public and carer involvement groups. (2015). Identifying Continence OptioNs after Stroke (ICONS): an evidence synthesis, case study and exploratory cluster randomised controlled trial of the introduction of a systematic voiding programme for patients with urinary incontinence after stroke in secondary care. NIHR Journals Library. | Individual interview | Acceptability/Feasibility   Adoption   Adaptation and evolution   Champion/Field agent   Compatibility   Context - Inner setting   Evaluation   Fit   Knowledge and knowledge synthesis   Implementation   Stakeholders   Sustainability | Implementation   Sustainment |
| i-PARIHS interview guide | Harvey G, Kitson A. PARIHS revisited: from heuristic to integrated framework for the successful implementation of knowledge into practice. Implement Sci. 2016;11:33. Published 2016 Mar 10. doi:10.1186/s13012-016-0398-2 Harvey G,  Kitson A. Implementing evidence-based practice in healthcare: a facilitation guide. London: Routledge; 2015. | Individual interview | Adaptation and evolution Adopter/Implementer/Decision maker characteristics   Barriers and facilitators   Context - Inner setting   Context - Outer setting   Knowledge and knowledge synthesis   Patient/Target audience characteristics and needs  Strategies | Implementation |
| PRISM interview guide | Ayele, Roman A., et al. "Study protocol: improving the transition of care from a non-network hospital back to the patient’s medical home." BMC health services research 17 (2017): 1-8. | Individual interview | Context – Inner settings   Context – Outer setting   Evaluation   Patient/target audience characteristics and needs   Pre-implementation   Stakeholders   Sustainability | Pre-Implementation   Implementation |
| CFIR interview guide (Lam) | Lam H, Quinn M, Cipriano-Steffens T, Jayaprakash M, Koebnick E, Randal F, Liebovitz D, Polite B, Kim K. Identifying actionable strategies: using Consolidated Framework for Implementation Research (CFIR)-informed interviews to evaluate the implementation of a multilevel intervention to improve colorectal cancer screening. Implement Sci Commun. 2021 May 31;2(1):57. doi: 10.1186/s43058-021-00150-9. PMID: 34059156; PMCID: PMC8167995. | Individual interview | Barriers and facilitators   Evaluation   Innovation characteristics   Context - Inner setting   Context - Outer setting   Process  Stakeholder | Pre-Implementation   Implementation |
| Program Sustainability Index | Mancini, J. A., & Marek, L. I. (2004). Sustaining Community-Based Programs for Families: Conceptualization and Measurement. Family Relations: An Interdisciplinary Journal of Applied Family Studies, 53(4), 339–347. <https://doi.org/10.1111/j.0197-6664.2004.00040.x>    Marek, L. I., Mancini, J. A., & Brock, D. J. (2003). National State Strengthening Program Sustainability Study: Patterns of early sustainability. Blacksburg, VA: Virginia, 2033. | Survey | Sustainability | Sustainment |
| Partnership/synergy assessment tool | Weiss, Elisa S., Rebecca Miller Anderson, and Roz D. Lasker. "Making the most of collaboration: exploring the relationship between partnership synergy and partnership functioning." Health Education & Behavior 29.6 (2002): 683-698. | Survey | Barriers and facilitators   Champion/Field agent  Context - Inner setting Context - Outer setting   Cost   Engagement   Knowledge and knowledge synthesis   Knowledge transfer and utilization  Evaluation   Reach   Stakeholders   Strategies | Pre-Implementation   Implementation   Sustainment |
| Context Matters Reporting Template | Stange KC and Glasgow RE. Considering and Reporting Important Contextual Factors in Research on the Patient-Centered Medical Home. Rockville, MD: Agency for Healthcare Research and Quality. May 2013. ARHQ Publication No. 13-0045-EF. | Other (Reporting template) | Context   Context - Inner setting   Context - Outer setting | Pre-Implementation   Implementation   Sustainment |
| Ethnographic assessment of partner engagement | Rabin, B.A., Cain, K.L., Salgin, L. *et al.* Using ethnographic approaches to document, evaluate, and facilitate virtual community-engaged implementation research. *BMC Public Health* **23**, 409 (2023). <https://doi.org/10.1186/s12889-023-15299-2> | Observation guide Other (Ethnographic assessment) | Engagement | Pre-Implementation   Implementation   Sustainment |
| Short PSAT | Luke DA, Calhoun A, Robichaux CB, Elliott MB, Moreland-Russell S. The Program Sustainability Assessment Tool: a new instrument for public health programs. Prev Chronic Dis. 2014 Jan 23;11:130184. doi: 10.5888/pcd11.130184. PMID: 24456645; PMCID: PMC3900326. | Survey | Adaptation and evolution   Communication   Context  Context – Inner setting   Context – Outer setting   Cost   Dissemination  Engagement   Evaluation | Sustainment |
| Research Engagement Survey Tool - 9 item | Goodman, M.S.; Ackermann, N.; Pierce, K.A.; Bowen, D.J.; Thompson, V.S. Development and Validation of a Brief Version of the Research Engagement Survey Tool. Int. J. Environ. Res. Public Health 2021, 181, 20. <https://doi.org/10.3390/ijerph181910020> | Survey | Engagement   Health equity   Stakeholder | Pre-Implementation   Implementation   Sustainment |
| Local Wellness Policy Implementation Checklist | Probart C, McDonnell ET, Jomaa L, Fekete V. Lessons from Pennsylvania’s mixed response to federal school wellness law. 2010;29(3):477-453. <https://doi.org/10.1377/hlthaff.2009.0732> | Survey | Champion/Field agent   Communication channels   Context - Inner setting   Context - Outer setting   Engagement   Evaluation   Implementation   Knowledge and knowledge synthesis   Outcomes - Implementation   Reach  Stakeholders   Strategies | Pre-Implementation   Implementation |
| Local Wellness Policy Survey | McDonnell E, Probart C. School wellness policies: employee participation in the development process and perceptions of the policies. The Journal of Child Nutrition & Management. 2008;32(1). <https://schoolnutrition.org/5--News-and-Publications/4--The-Journal-of-Child-Nutrition-and-Management/Spring-2008/Volume-32,-Issue-1,-Spring-2008---McDonnell;Probart/> | Survey | Development of an intervention   Pre-Implementation   Process | Pre-Implementation |
| Rehabilitation Policy Questionnaire | Brämberg EB, Klinga C, Jensen I, et al. Implementation of evidence-based rehabilitation for non-specific back pain and common mental health problems: a process evaluation of a nationwide initiative. BMC Health Services Research. 2015;15(1):1-11. doi:10.1186/s12913-015-0740-4. | Survey | Barriers and facilitators   Context - Inner setting   Context - Outer setting   Innovation characteristics   Adopter/Implementer/Decision maker characteristics | Implementation |
| Diagnosis Related Group Policy Survey | Tummers LG, Van de Walle S. Explaining health care professionals’ resistance to implement Diagnosis Related Groups: (No) benefits for society, patients and professionals. Health Policy. 2012;108(2/3):158-166. Doi.org/10.1016/j.healthpol.2012.08.024    Tummers LG, Van de Walle S. Policy Implementation, Street-level bureaucracy, and the importance of discretion. Public Management Review. 2014;16(4):527–547. doi.10.1080/14719037.2013.841978 | Survey | Acceptability/Feasibility   Adoption   Fit   Outcomes - Quality improvement/Practice or policy change | Pre-Implementation |
| Rütten’s Health Policy Questionnaire | Rütten A, Lüschen G, von Lengerke T, et al. (2003). Determinants of health policy impact: comparative results of a European policymaker study. Sozial- Und Praventivmedizin, 48(6), 379–391. doi: 10.1007/s00038-003-2048-0 | Survey | Context - Outer setting   Outcomes - Quality improvement/Practice or policy change | Implementation   Sustainment |
| Policy Coalition Evaluation Tool | Hardy LJ, Wertheim P, Bohan K, Quezada JC, Henley E. A model for evaluating the activities of a coalition-based policy action group: the case of Hermosa Vida. Health Promotion Practice. 2013;14(4):514-523. doi:10.1177/1524839912461253 | Documentation guide | Context - Inner setting   Context - Outer setting   Fit   Identification   Implementation   Innovation characteristics   Sustainability   Outcomes - Quality improvement/Practice or policy change   Process   Stakeholders | Implementation   Sustainment |
| CFIR Interview Guide (Zhao) | Zhao, X., Varisco, R., Borghouts, J. et al. Facilitators of and barriers to County Behavioral Health System Transformation and Innovation: an interview study. BMC Health Serv Res 24, 604 (2024). <https://doi.org/10.1186/s12913-024-11041-9> | Individual interview   Focus group interview | Adopter/Implementer/Decision maker characteristics   Barriers and facilitators   Evaluation   Innovation characteristics   Context - Inner setting   Context - Outer setting   Process   Stakeholder | Pre-Implementation Implementation |
| CFIR Interview Guide Webtool | Original: Damschroder, L.J., Aron, D.C., Keith, R.E. et al. Fostering implementation of health services research findings into practice: a consolidated framework for advancing implementation science. Implementation Sci 4, 50 (2009). <https://doi.org/10.1186/1748-5908-4-50>    Updated: Damschroder LJ, Reardon CM, Widerquist MAO, Lowery J. The updated Consolidated Framework for Implementation Research based on user feedback. Implement Sci. 2022 Oct 29;17(1):75. doi: 10.1186/s13012-022-01245-0. PMID: 36309746; PMCID: PMC9617234. | Individual interview   Focus group interview | Adaptation and evolution Adopter/Implementer/Decision maker characteristics  Barriers and facilitators   Compatibility   Context - Inner setting   Context - Outer setting   Cost   Engagement   Evaluation   Innovation characteristics   Outcomes  Process   Readiness   Relative advantage   Stakeholder   Strategies | Pre-Implementation   Implementation |
| PRISM Contextual Survey Instrument (PCSI) | Pittman, J.O.E., Almklov, E., Rabin, B., Gault, J., Blanco, B., Lindamer, L. (2023, December 10-13). Development of a pragmatic measure for the practical, robust implementation and sustainability model (PRISM) [Presentation]. Conference on the Science of Dissemination and Implementation in Health, Arlington, VA. | Survey | Acceptability/Feasibility Adopter/Implementer/Decision maker characteristics  Adoption   Barriers and facilitators  Context   Context – Inner setting   Context – Outer setting  Fit   Implementation   Sustainability   Patient/Target audience characteristics and needs   Pre-Implementation   Readiness | Pre-Implementation |
| Implementation Strategy Usability Scale | Lyon, A.R., Coifman, J., Cook, H. *et al.* The Cognitive Walkthrough for Implementation Strategies (CWIS): a pragmatic method for assessing implementation strategy usability. *Implement Sci Commun* **2**, 78 (2021). <https://doi.org/10.1186/s43058-021-00183-0> | Survey | Acceptability/Feasibility Adopter/Implementer/Decision maker characteristics   Implementation   Strategies | Implementation |
| Provider REport of Sustainment Scale (PRESS) | Moullin, J. C., Sklar, M., Ehrhart, M. G., Green, A., & Aarons, G. A. (2021). Provider REport of Sustainment Scale (PRESS): development and validation of a brief measure of inner context sustainment. Implementation Science, 16(1). <https://doi.org/10.1186/s13012-021-01152-w> | Survey | Sustainability | Sustainment |
| Readiness Thinking Tool - Survey | Using readiness. Wandersman Center. (n.d.). <https://www.wandersmancenter.org/using-readiness.html> | Survey | Acceptability/feasibility   Adoption   Barriers and facilitators   Champion/Field agent   Communication   Communication channels   Compatibility   Complexity   Context   Context – Inner setting   Context – Outer setting   Cost  Implementation   Innovation characteristics   Knowledge and knowledge synthesis   Observability   Pre-implementation   Readiness | Pre-Implementation |
| Readiness for Recovery and Resiliency-Interview Guide | Using readiness. Wandersman Center. (n.d.). <https://www.wandersmancenter.org/using-readiness.html> | Focus group interview | Acceptability/Feasibility   Adoption   Barriers and facilitators   Champion/Field agent   Communication   Communication channels   Compatibility   Complexity   Context   Context – Inner setting   Context – Outer setting   Cost  Implementation   Innovation characteristics   Knowledge and knowledge synthesis   Observability   Pre-implementation   Readiness | Pre-Implementation |
| User Engagement Scale - Short | O'Brien, H.L. and Toms, E.G. (2010), The development and evaluation of a survey to measure user engagement. J. Am. Soc. Inf. Sci., 61: 50-69. <https://doi.org/10.1002/asi.21229>    O’Brien, Heather L., et al. “A Practical Approach to Measuring User Engagement with the Refined User Engagement Scale (UES) and New UES Short Form.” International Journal of Human-Computer Studies, vol. 112, Apr. 2018, pp. 28–39, <https://doi.org/10.1016/j.ijhcs.2018.01.004> | Survey | Engagement | Not found |
